# An *in vitro* Study on the Role of Hepatitis B Virus X Protein C-Terminal Truncation in Liver Disease Development

**DOI:** 10.3389/fgene.2021.633341

**Published:** 2021-03-12

**Authors:** Zaheenul Islam Siddiqui, Syed Ali Azam, Wajihul Hasan Khan, Masarrat Afroz, Sabihur Rahman Farooqui, Fatima Amir, Md Iqbal Azmi, Ayesha Anwer, Saniya Khan, Mahboubeh Mehmankhah, Shama Parveen, Syed Naqui Kazim

**Affiliations:** ^1^Centre for Interdisciplinary Research in Basic Sciences, Jamia Millia Islamia, New Delhi, India; ^2^Department of Microbiology, College of Medicine, Imam Abdulrahman Bin Faisal University, Dammam, Saudi Arabia

**Keywords:** hepatitis B virus X protein mutation, HBx C-terminal truncation, HBx C-terminal deletion, liver disease development, hepatocellular carcinoma, cirrhosis, fibrosis, HBx expression pattern

## Abstract

Hepatitis B virus X protein C-terminal 127 amino acid truncation is often found expressed in hepatocellular carcinoma (HCC) tissue samples. The present *in vitro* study tried to determine the role of this truncation mutant in the hepatitis B–related liver diseases such as fibrosis, cirrhosis, HCC, and metastasis. HBx gene and its 127 amino acid truncation mutant were cloned in mammalian expression vectors and transfected in human hepatoma cell line. Changes in cell growth/proliferation, cell cycle phase distribution, expression of cell cycle regulatory genes, mitochondrial depolarization, and intracellular reactive oxygen species (ROS) level were analyzed. Green fluorescent protein (GFP)–tagged version of HBx and the truncation mutant were also created and the effects of truncation on HBx intracellular expression pattern and localization were studied. Effect of time lapse on protein expression pattern was also analyzed. The truncation mutant of HBx is more efficient in inducing cell proliferation, and causes more ROS production and less mitochondrial depolarization as compared with wild type (wt) HBx. In addition, gene expression is altered in favor of carcinogenesis in the presence of the truncation mutant. Furthermore, mitochondrial perinuclear aggregation is achieved earlier in the presence of the truncation mutant. Therefore, HBx C-terminal 127 amino acid truncation might be playing important roles in the development of hepatitis B–related liver diseases by inducing cell proliferation, altering gene expression, altering mitochondrial potential, inducing mitochondrial clustering and oxidative stress, and changing HBx expression pattern.

## Introduction

**FIGURE 1:**
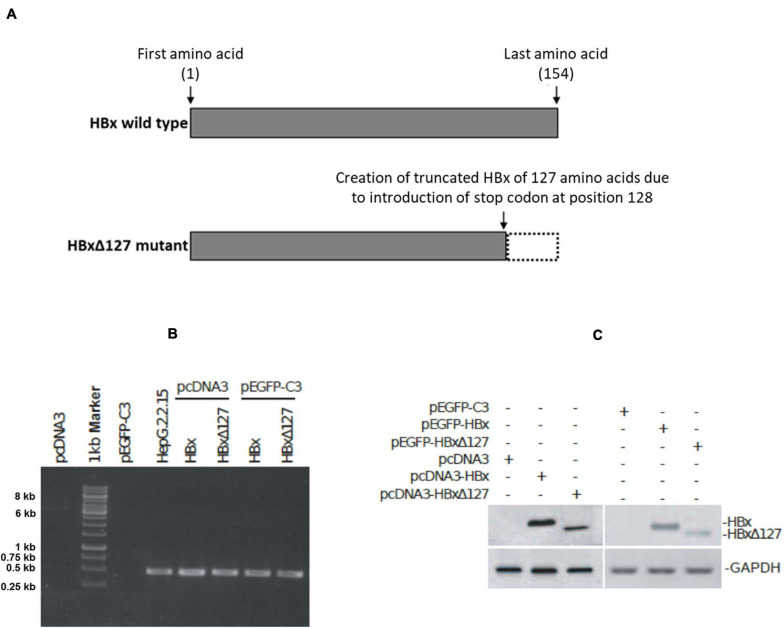
Expression of HBV X protein (HBx) and HBxΔ127 from pcDNA3 and pEGFP-C3 constructs. **(A)** Full-length HBV X protein (HBx) and C-terminal 27 amino acid deletion mutant HBxΔ127. **(B)** RT-PCR product analyzed by gel electrophoresis on 1% agarose. PCR was performed using cDNA samples originating from cells transfected with empty-pcDNA3, empty-pEGFP-C3, pcDNA3-HBx, pcDNA3-HBxΔ127, pEGFP-C3-HBx, and pEGFP-C3-HBxΔ127, and HBx-specific primers. cDNA originating from HepG2.2.15 cells was used as positive control. cDNA originating from cells transfected with empty-pcDNA3 and pEGFP-C3 were used as negative controls. HBx gene (465 bp long) corresponding to 0.5-kb band of marker was successfully amplified from cells transfected with pcDNA3-HBx, pcDNA3-HBxΔ127, pEGFP-C3-HBx, and pEGFP-C3-HBxΔ127. HBx was also amplified from HepG2.2.15 cells. No amplification was observed from empty-pcDNA3 and empty-pEGFP-C3 transfected cells. **(C)** Western blot. HBx-specific bands observed for protein samples originating from cells transfected with pcDNA3-HBx, pcDNA3-HBxΔ127, pEGFP-C3-HBx, and pEGFP-C3-HBxΔ127. No HBx band was observed for protein samples isolated from empty-pcDNA3- and empty-pEGFP-C3-transfected cells. GAPDH was used as loading control.

Nearly 350 to 400 million people are chronically infected with hepatitis B virus (HBV) worldwide (Kao et al., 2000). Chronic carriers of HBV have multifold higher risk of developing liver cirrhosis and hepatocellular carcinoma (HCC) than uninfected patients (Feitelson et al., 2005). The role of HBV X protein (HBx) is widely studied for its involvement in development of liver diseases, specifically HCC (Yu et al., 1999; Kew, 2011). High rates of mutations are reported in the viral genomes isolated from chronic HBV patients (Kao et al., 2000). Different lengths of HBV DNA integrated in host genome are also common among these patients (Yang et al., 2018). It is estimated that around 80 to 90% of chromosomal DNA isolated from hepatitis B–related HCC patients have integrated HBV fragments (Tu et al., 2001; Wang et al., 2004). Some of these random mutations and integrations cause creation of stop codons, which result in expression of truncated versions of HBV proteins, specifically HBx at its c terminus (Tu et al., 2001; Wang et al., 2004; Caligiuri et al., 2016). Various C-terminal deleted (truncated) mutants of HBx are expressed in HBV-infected and related cirrhosis and HCC patients’ samples (Ma et al., 2008). Furthermore, the C-terminal truncated version of HBx are reported by several research groups to be overexpressed in HBV-related HCC tissues (Sirma et al., 1999; Tu et al., 2001; Iavarone et al., 2003; Ma et al., 2008). One such truncated version of HBx commonly found in these patients is 127 amino acid C-terminal deletion mutant (HBxΔ127) resulting from creation of a stop codon at 128th amino acid position of HBx (Figure 1A). It is reported that HBxΔ127 favors proliferation of host cells and may be involved in HCC development (Wang et al., 2010). Mutated versions of HBx function quite differently from its wild-type (wt) counterpart and are involved in development of liver diseases, specifically HCC (Hagiwara et al., 2017). A recent study revealed a specific role of natural C-terminal truncated HBx in inducing cancer stem cell, tumor-initiating properties, and drug resistance in the host cells. However, the mechanism underlying these changes remains unclear (Ching et al., 2017). Another study showed that C-terminal truncated HBx contributes in HCC development, at least in part, by regulating miRNA expression (Yip et al., 2011). The present *in vitro* study tried to determine the roles of HBxΔ127 at cellular and molecular levels in the development of liver diseases after chronic HBV infection. For this, HBx gene and its truncation mutant HBxΔ127 were cloned in mammalian expression vectors, pcDNA3 and pEGFP-C3, and the constructs were transfected in huh7 cell line. The effect of HBxΔ127 on cell growth/proliferation, gene expression, intracellular reactive oxygen species (ROS) level, mitochondrial potential, and HBx gene expression were studied.

## Materials and Methods

### Cloning HBx and Site-Directed Mutagenesis (SDM)

The 465-bp HBx coding DNA was PCR amplified from a plasmid pHBV harboring 1.3 times the HBV genome (serotype-ayw1, genotype D). Primers HBx-*Kpn*I-F and HBx-*Xho*I-R (Supplementary Materials) with overhangs for restriction enzyme recognition sites were used for amplification. High-fidelity Taq DNA polymerase (Thermo Scientific, MA, United States) was used in PCR. The thermal conditions were as follows: initial denaturation at 95°C for 5 min; 30 cycles of denaturation at 95°C for 30 s, annealing at 54°C for 30 s, extension at 72°C for 30 s; and final extension at 72°C, 5 min. The amplified product was gel purified using Sure Extract PCR Clean-up/Gel Extraction Kit (Genetix Biotech, New Delhi, India). The purified PCR product was then ligated using T4 DNA ligase (Thermo Scientific) into a cloning vector pTZ57R/T (a part of InsTAclone PCR Cloning Kit; Thermo Scientific). Competent *Escherichia coli* DH5α cells were transformed with the ligation product and successful clone was screened by blue white screening and colony PCR. The successful construct was named pTZ57R/T-HBx.

#### Subcloning HBx in pcDNA3

pTZ57R/T-HBx and pcDNA3 (Invitrogen, CA, United States) were treated with restriction enzymes *Kpn*I and *Xho*I (Thermo Scientific). Restricted out HBx and linearized pcDNA3 were gel purified, and then ligated using T4 DNA ligase. The ligated product was transformed in the competent cells as mentioned previously and the successful clone was screened by colony PCR. The construct was named pcDNA3-HBx.

#### SDM

The C-terminal 27 amino acids of HBx were deleted to create HBx 127 amino acid truncation mutant (HBxΔ127, Figure 1A) by introducing a stop codon at the 128th amino acid position of HBx. For this, PCR of 18 cycles was performed using pcDNA3-HBx, SDM primers, and high-fidelity Taq DNA polymerase. The PCR product was treated with *Dpn*I (Thermo scientific) and transformed in *E. coli* JM109 competent cells. The resulting mutant was named pcDNA3-HBxΔ127. The constructs, pcDNA3-HBx and pcDNA3-HBxΔ127, were confirmed for proper integration sites and successful mutation, respectively, by sequencing by Xcelris Genomics (Gujarat, India) using T7 promoter forward primer and BGH reverse primer.

#### Subcloning in pEGFP-C3

After confirming expression of HBx and HBxΔ127 from pcDNA3-HBx and pcDNA3-HBxΔ127 in Huh7 cells by RT-PCR and western blot (detailed later), both versions of HBx were cloned in pEGFP-C3. For this, pcDNA3-HBx, pcDNA3-HBxΔ127, and pEGFP-C3 were restricted with *Hin*dIII and *Apa*I (Thermo Scientific). The linearized pEGFP-C3 and restricted out HBx and HBxΔ127 were ligated using T4 DNA ligase in separate reactions. The ligation products were transformed in JM109 competent cells. The GFP-fusion clones were confirmed by sequencing with EGFP C primer, and named pEGFP-C3-HBx and pEGFP-C3-HBxΔ127. Sequences of all the primers mentioned and the sequencing results can be found in Supplementary Materials. All the primers were manufactured by Xcelris Genomics. The HBx expression by cytomegalovirus promoter-enhancer, used by pcDNA3 and pEGFP-C3, is reported to be within the physiological limits of HBV-infected cells (Su and Schneider, 1997; Tanaka et al., 2004, Supplementary Materials).

### Cell Culture and Transient Transfection

Human hepatoma cell line Huh7 was cultured in Dulbecco’s Modified Eagle’s Medium, 10% fetal bovine serum, and penicillin–streptomycin (100 μg/ml each) (Genetix Biotech) in an incubator at 37°C in a humid atmosphere with 5% CO_2_. Lipofectamine 2,000 (Invitrogen) was used for transfection of plasmid constructs in the cells according to the manufacturer’s instructions. pcDNA3 constructs (0.2, 0.5, 1, and 1.5 μg) were used for transfection in 96-, 24-, 12-, and 6-well culture plates, respectively. Empty pcDNA3 transfected cells served as control for all experiments, except those involving GFP-HBx fusion proteins, where empty pEGFP-C3 transfected cells served as control. HepG2.2.15, a cell line with integrated HBV genome in its chromosomal DNA, was also cultured. Complementary DNA (cDNA) originating from HepG2.2.15 was used as positive control in RT-PCR experiments. Transfection efficiency was 60–70%, as monitored by GFP expression, by co-transfecting cells (in a 6-well plate) with 0.5 μg pEGFP-C3. Huh 7 cell line is selected for this study because it is extensively used as an *in vitro* system by the scientific community to study liver diseases, specifically HCC (reviewed in Supplementary Materials).

### Reverse Transcriptase-PCR

Huh7 cells (50,000/well) were seeded in a 24-well plate, and after cells reached 70% confluency, they were transfected with empty pcDNA3, empty-pEGFP-C3, pcDNA3-HBx, pcDNA3-HBxΔ127, pEGFP-C3-HBx, and pEGFP-C3-HBxΔ127. Total cellular RNA was isolated using TRIzol reagent (Invitrogen), and 1 μg RNA was used for cDNA synthesis using oligo (dT)_18_ primers, reverse transcriptase (RT), and RNase inhibitor (Thermo Scientific). Thermal conditions for cDNA synthesis were 42°C for 60 min (reverse transcription) followed by heat inactivation at 70°C for 15 min. RNA was also isolated from HepG2.2.15 cells, and cDNA was synthesized for use as positive control. RNA isolation and cDNA synthesis were performed according to the manufacturer’s instructions. PCR was done using 0.5 μl of cDNA as template and HBx-specific primers (Supplementary Materials). Thermal conditions for PCR were same as mentioned for HBx amplification for cloning.

### Western Blotting

Cells (250,000/well) were seeded in a 6-well plate and after cells reached 70% confluency, they were transfected with empty-pcDNA3, pcDNA3-HBx, pcDNA3-HBxΔ127, empty-pEGFP-C3, pEGFP-C3-HBx, and pEGFP-C3-HBxΔ127. Cells were lysed using RIPA buffer (Thermo Scientific) with addition of 50 mM sodium fluoride, 1 mM sodium orthovanadate, 2 μg/ml aprotinin (Sigma-Aldrich, United States), and 1 mM phenylmethylsulfonyl fluoride (Sigma-Aldrich). Concentration of isolated protein samples was determined by Protein Estimation Kit by BCA Method (GeNei, Bangalore, India). Protein samples were denatured by adding 1 × Laemmli buffer (Amresco, United States) and then heating at 95°C for 15 min. Equal amounts of proteins were subjected to 4–12% sodium dodecyl sulfate polyacrylamide (Sigma-Aldrich) gel electrophoresis (SDS-PAGE). The bands on the gel were then transferred on nitrocellulose membrane (BioTrace NT; Pall Corporation, NY, United States). The membrane was blocked in PBS with 5% bovine serum albumin (G-Biosciences, United States) and 0.05% Tween-20 (Merck, NJ, United States). Western blots were analyzed using mouse IgG1 antibodies against HBx (primary antibody, dilution 1:250; Santa Cruz Biotechnology, TX, United States) and GAPDH (primary antibody, dilution 1:1,000; Thermo Scientific), HRP-linked antibody anti-mouse IgG (secondary antibody, dilution 1:10,000) (Santa Cruz Biotechnology), and enhanced chemiluminescence reagents (Thermo Scientific). The membrane was treated overnight with primary antibodies at 4°C and then with secondary antibody for 2 h at room temperature. The electrophoresis set-up was from Bio-Rad, United States.

### MTT Assay

3-(4,5-Dimethylthiazol-2-yl)-2,5-diphenyltetrazolium bromide (MTT; Sigma-Aldrich) assay was performed as described previously (Rastegar et al., 2010). Cells were seeded in a 96-well plate, allowed to attach overnight and transfected. Culture was maintained further for 4 days. For the MTT assay, the culture media in each of the culture wells was replaced with 100 μl of 1 mg/ml MTT solution. The plate was incubated for 4 h at 37°C. After the required incubation period, plate was centrifuged at 3,000 rpm for 10 min and supernatant was discarded. Afterward, 50 μl dimethyl sulfoxide (Sigma-Aldrich) was added in each well. The plate was incubated at room temperature for 15 min. After incubation, optical density (OD) at 570 nm was taken using an ELISA microplate reader (Bio-Rad). Relative OD values were calculated using values obtained for empty-pcDNA3 transfected wells as reference. All the incubations were done on a rocker in dark.

### Flow Cytometry

Cell cycle experiments were performed as previously described (Mukherji et al., 2007). Cells were harvested 48 h after transfection and centrifuged at 800 rpm. The pellet was washed with phosphate-buffered saline (PBS) and again resuspended in 300 μl PBS. Cells were fixed by adding 700 μl of ice-cold 70% ethanol in a dropwise manner and incubating in ice for 1 h. Ethanol was then removed by centrifugation of the cell suspension at 1,000 rpm for 10 min. The cell pellet was washed in PBS, the pellet resuspended in 1 ml of 0.1 μg/ml RNaseA (Merck) solution, and incubated at 37°C for 1 h. For staining the cells for flow cytometry analysis, 10 μl of propidium iodide (Merck) stock solution (1 mg/ml) was added to the cell suspension to make overall PI concentration of 10 μg/ml in the cell suspension. The cells were incubated at 37°C for 1 h. Cell cycle study was performed at 488 nm using a flow cytometer (BD Biosciences, CA, United States) and retrieved data analyzed by FlowJo.

### Cell Growth Assay

Cell growth assay performed as described previously (Rastegar et al., 2010; Cheong et al., 2011; Guzman et al., 2014). For this, cells were harvested 36 h post-transfection and re-plated in fresh wells. Cells were cultured for five more days and afterward stained with 0.2% crystal violet (Sigma-Aldrich) solution for 15 min at room temperature. For removing the unbound stain, the wells were washed three times with PBS. The cell growth/colony formation was observed using a camera (Canon IXUS; Canon, China). Afterward, the cells in the culture wells were dried by placing the culture plates inverted on a tissue paper towel for 5 min. Cell-bound crystal violet was retrieved by adding to the culture wells 100 μl 0.1% SDS (Sigma-Aldrich) solution. The plate was incubated for 5 min and the SDS solution retrieved from culture wells. The dissolved crystal violet in the retrieved SDS solution was quantified by taking OD at 570 nm. All the staining and washing steps were performed in dark on a rocker.

### Real-Time qRT-PCR

Expression levels of CDK2, B-Myb, C-Myb, E2F1, p21^Cip1^, and p27^Kip1^ genes were analyzed by real-time PCR (StepOnePlus; Applied Biosystems, CA, United States) using SYBR Green I (Applied Biosystems), cDNA (from cells 48 h post-transfection), and gene-specific primers (Supplementary Materials). Relative quantification values calculated by 2^–ΔΔCt^ method as described previously (Livak and Schmittgen, 2001). GAPDH was used for normalization of input cDNA. Thermal conditions for amplification were initial denaturation for 10 min at 95°C followed by 40 cycles of denaturation for 25 s at 95°C, annealing for 25 s (at temperatures mentioned in Supplementary Materials along with each primer), and extension for 25 s at 72°C. For each sample, total reaction mixture of 5 μl was set in triplicate, using 0.25 μl cDNA as template. Melt curve analysis for the amplified products was performed according to the instrument’s default thermal conditions.

### Tetramethylrhodamine Ethyl Ester Staining

The tetramethylrhodamine ethyl ester (TMRE) staining was performed as described previously (Gaspar et al., 2006). The working TMRE (Sigma-Aldrich) solution (200 nM) was made in culture media. Forty-eight hours after the transfection experiments, the culture media was removed from the wells and 50 μl of the working TMRE solution was added in each well. The plate was incubated for 30 min at 37°C. To remove excess stain, culture wells were washed with a solution of 0.2% BSA and TMRE fluorescence measured by plate reader. The BSA solution used for washing was made in 1 × PBS. All the staining and washing steps were performed in dark. Data were expressed as percentage of intensity of control culture as%TMRE fluorescence = (TMRE fluorescence_HBx/HBxΔ 127 transfected culture_ − TMRE fluorescence_blank_) × 100/(TMRE fluorescence_control culture_ − TMRE fluorescence_blank_).

### Dihydroethidium Staining

Dihydroethidium (DHE) (Sigma-Aldrich) is oxidized by intracellular ROS to form ethidium, which in turn intercalates with DNA inside the nucleus, giving nuclear staining to ROS-positive cells. DHE staining was performed as described previously (Gu et al., 2006). Forty-eight hours post-transfection, cells were stained for 30 min, in dark, with a solution of 10 μM DHE (Sigma-Aldrich) at 37°C.

### Direct Microscopic Observation of Intracellular Expression of GFP-HBx and GFP-HBxΔ127

Cells were seeded in a 24-well plate, and when growth reached 70% confluency, they were transfected with pEGFP-C3-HBx and pEGFP-C3-HBxΔ127. At different culture observation timepoints, starting from 18 to 96 h and more, culture wells were washed with PBS and cells treated with DAPI antifade mountant (Santa Cruz Biotechnology). Observations were made using an inverted fluorescence microscope (Nikon, Tokyo, Japan).

### Statistical Analysis

Each set of experiments was repeated independently for at least three times. Results were presented as means ± SD. Statistical significance was determined using paired two-tailed Student’s *t*-test. *P*-values of <0.05 were considered statistically significant.

## Results

### Intracellular Expression of HBx and HBxΔ127 From pcDNA3 and pEGFP-C3 Constructs

Amplicons of 465 bp were successfully PCR amplified using HBx-specific primers and cDNA from transfectants with pcDNA3-HBx, pcDNA3-HBxΔ127, pEGFP-C3-HBx, and pEGFP-C3-HBxΔ127 (Figure 1B). No amplification was observed from cDNA from control cells transfected with empty-pcDNA3 and empty-pEGFP-C3, and amplification was observed for cDNA derived from HepG2.2.15 (Figure 1B). Western blot results showed HBx-specific bands for whole cell lysates from transfectants with pcDNA3-HBx, pcDNA3-HBxΔ127, pEGFP-C3-HBx, and pEGFP-C3-HBxΔ127 (Figure 1C). No HBx-specific band was observed for lysates of control cells transfected with empty-pcDNA3 and empty-pEGFP-C3 (Figure 1C). GAPDH was used as control.

### HBxΔ127 Induces Cell Proliferation and Increases Percentage of Cells in S and G2/M Cell Cycle Phases

**FIGURE 2:**
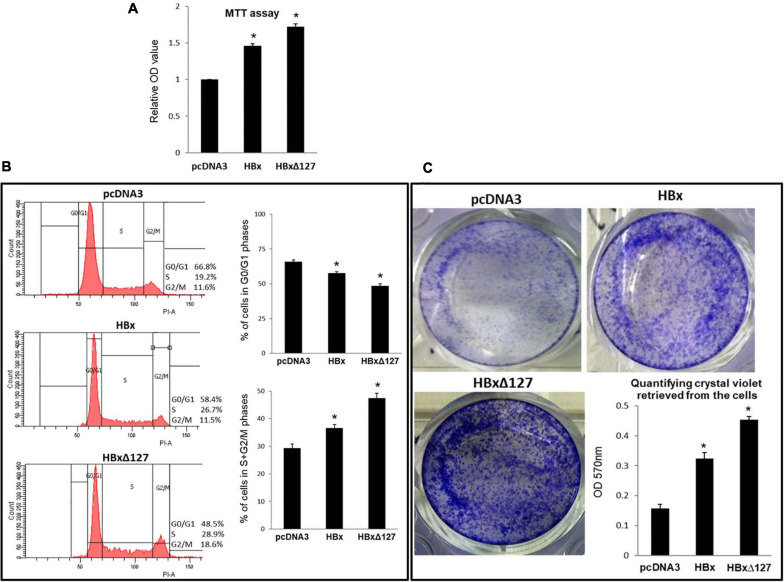
Cell growth and proliferation. **(A)** MTT assay. 10,000 cells/well were seeded in a 96-well plate and transfected with empty pcDNA3, pcDNA3-HBx, and pcDNA3-HBxΔ127. Cells were subjected to MTT assay 4 days post-transfection. Experiments were performed in dark and optical density (OD) at 570 nm taken using a microplate reader. Relative OD values were calculated using values obtained for empty-pcDNA3 transfected wells as reference. Data shown as mean ± SD of three independent experiments performed in quadruplicate. **(B)** Cell cycle analysis. Huh7 cells were seeded in a 6-well plate, and after the cells grew 70% confluent, the cells were transfected with empty pcDNA3, pcDNA3-HBx, and pcDNA3-HBxΔ127. Forty-eight hours post-transfection, cells were fixed in 70% ethanol, stained with propidium iodide, and analyzed using a flow cytometer. Histograms represent one of the three independent experiments. Bar graphs show percentage of cells in G0/G1, and S + G2/M phases of cell cycle for empty-pcDNA3-transfected cells (control), and HBx- and HBxΔ127-transfected cells. Bar graphs represent mean ± SD of three independent experiments performed in duplicate. **(C)** Cell growth assay. Cells (100,000/well) were seeded in a 12-well plate, and at 70% confluency, the cells were transfected with empty pcDNA3, pcDNA3-HBx, and pcDNA3-HBxΔ127. Cells were maintained in culture conditions for 36 h, then harvested and replated (8,000 cells/well) in a 12-well plate. Cells were allowed to grow for five more days in the new culture plate and then stained with crystal violet. Bar graph shows quantification of crystal violet in the stained cells. Crystal violet retrieved in 0.1% SDS solution was quantified by taking optical density (OD) at 570 nm using a spectrophotometer. Data are shown as mean ± SD of three independent experiments performed in duplicate. For **(A–C)** data are analyzed by paired two-tailed Student’s *t*-test; *P < 0.05 compared with the control.

MTT assay results demonstrated higher cell proliferation in HBx-transfected cells as compared with control cells. When comparison was made between wtHBx and HBxΔ127, the latter was found to induce higher cell proliferation (Figure 2A). To investigate further on the effect of truncation mutation on host cell proliferation, flow cytometry of transfected cells was performed. The analysis revealed a decrease in percentage of cells in G0/G1 phases and a corresponding increase in percentage of cells in S and G2/M phases, in wtHBx-transfected cell cultures (Figure 2B). On comparing cells transfected with wtHBx and HBxΔ127, it was observed that the decrease in percentage of cells in G0/G1 phases and proportionate increase in percentage of cells in S and G2/M phases is more pronounced in the presence of HBxΔ127. The presence of truncation mutant resulted in an increase of 18% of cell population to proliferating phases (S and G2/M) as compared with around 7% increase in presence of wtHBx.

### HBxΔ127 Induces Cell Growth and Colony Formation

Higher growth and colony formation was observed for cells transfected with wtHBx and HBxΔ127 as compared with control (Figure 2C). When comparisons were made between the two versions of HBx, higher growth and colony formation was observed in cell cultures transfected with HBxΔ127. Correspondingly, higher amount of crystal violet was retrieved from HBxΔ127-transfected cultures (Figure 2C).

### HBxΔ127 Induces Expression of Cell Growth/Proliferation Linked Genes

**FIGURE 3:**
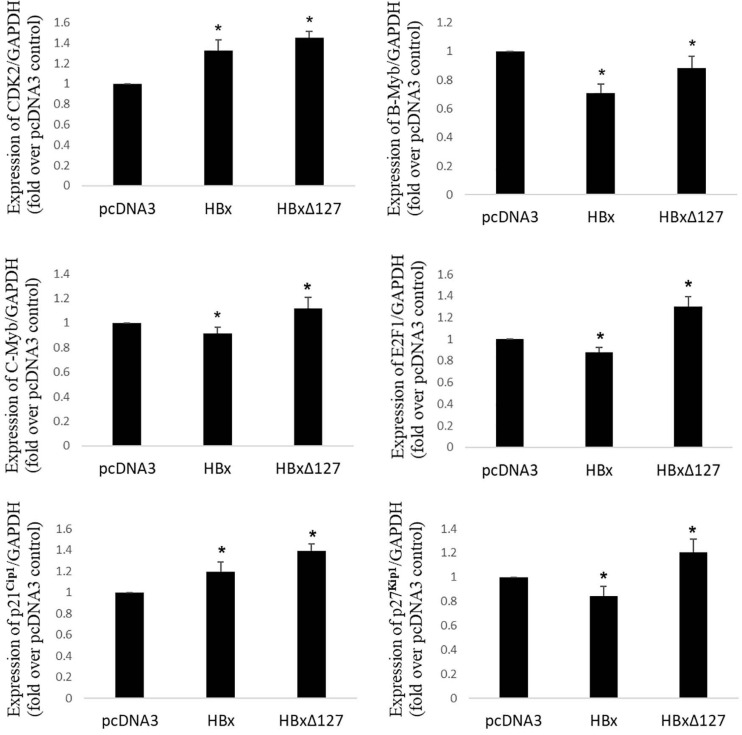
Real-time RT-PCR. Expression profile of important genes involved in control of cell growth/proliferation in cells transfected with empty pcDNA3, pcDNA3-HBx, and pcDNA3-HBxΔ127. GAPDH was used as internal control for each target gene. Relative quantification value for each target gene in the presence of pcDNA3 set as 1. Graphs showing mean ± SD of three independent experiments performed in triplicate. Data analysis by paired two-tailed Student’s *t*-test; *P < 0.05 compared with the value for pcDNA3-transfected cells.

To understand the molecular basis for higher cell growth/proliferation in presence of HBxΔ127, the expression of genes that are widely reported to control cell growth/proliferation, especially in the context of HBV-related liver disease development, was studied (please refer to the Discussion section for details). Out of 12 genes that were originally studied, noteworthy changes in gene expression in presence of HBxΔ127, as compared with wtHBx, were observed for CDK2, B-Myb, C-Myb, E2F1, p21^Cip1^, and p27^Kip1^ (Figure 3). Expression of genes CDK2, B-Myb, C-Myb, E2F1, p21^Cip1^, and p27^Kip1^ were higher in HBxΔ127-transfected cells as compared with wtHBx-transfected cells (Figure 3). Changes in expression pattern were more pronounced for E2F1 and p27^Kip1^. Expression of E2F1 was more than 40% higher, and that of p27^Kip1^ was 40% higher in the presence of HBxΔ127 as compared with wtHBx.

### HBxΔ127 Induces Mitochondrial Polarization

**FIGURE 4:**
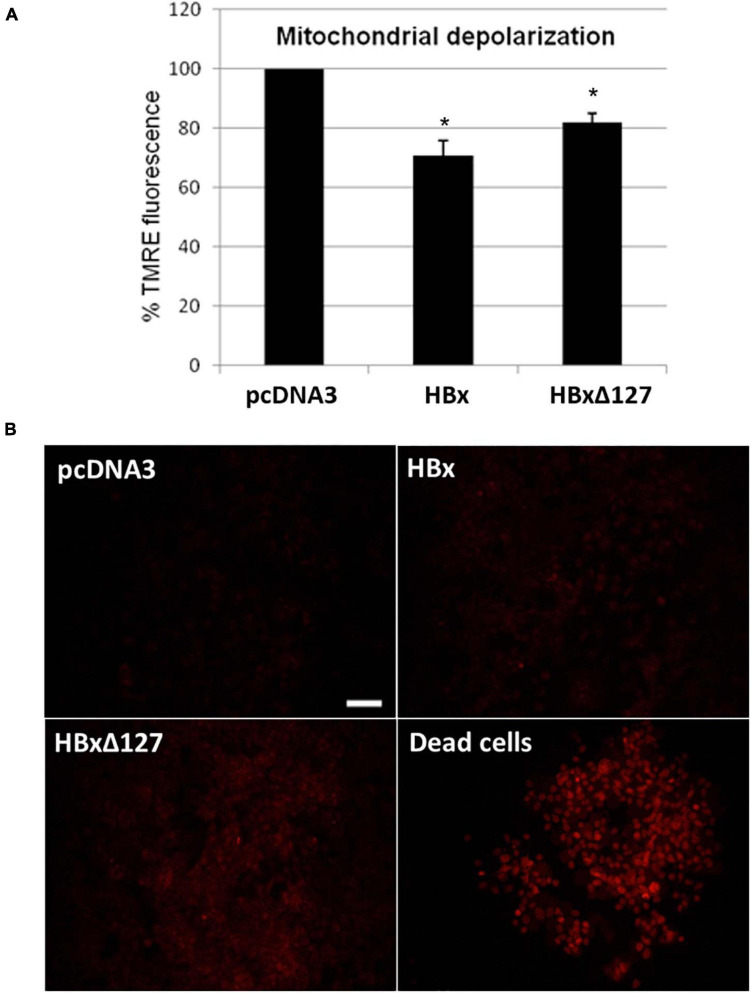
**(A)** Mitochondrial depolarization studied by tetramethylrhodamine ethyl ester (TMRE) staining. 10,000 cells/well were seeded in a 96-well plate and transfected with empty pcDNA3, pcDNA3-HBx, and pcDNA3-HBxΔ127. Forty-eight hours after transfection, cells were treated with TMRE and fluorescence measured by a microplate reader (Varioskan Flash Multimode Reader; Thermo Scientific) at excitation wavelength 549 nm and emission wavelength 575 nm. **(B)** Reactive oxygen species (ROS) production studied by dihydroethidium (DHE) staining. Huh7 cells cultured in a 12-well plate and transfected with empty pcDNA3, pcDNA3-HBx, and pcDNA3-HBxΔ127. Intracellular ROS formation was checked 48 h after transfection by treatment with DHE stain. Cells take DHE stain in proportion to their intracellular ROS level. As a positive control, dead cells prepared by prolonged media starvation were used. All the dead cells were 100% DHE positive. Empty-pcDNA3-transfected control cells used as negative control. Almost nil staining was observed for negative control. Significant increase in percentage of DHE-positive cells was observed in cell cultures transfected with HB×Δ127 as compared with wtHBx. Images were taken by fluorescent inverted microscope, 10× objective (Nikon, Tokyo, Japan). Scale bar, 100 μm. Identical acquisition parameters were used while taking these images. Images shown here represent one of the three independent experiments performed in triplicate.

Mitochondrial staining with TMRE is based on the fact that healthy polarized mitochondria stain bright red orange, whereas depolarized mitochondria of stressed/apoptotic cells result in decreased or nil staining (Lopez-Erauskin et al., 2012). Results showed that HBx induced mitochondrial depolarization. The depolarizing effect is less pronounced for HBxΔ127 than wtHBx (Figure 4A). This reflects more healthy and proliferating cells in presence of HBxΔ127 as compared with wtHBx.

### HBxΔ127 Induces Intracellular ROS Production

Dihydroethidium staining, which reflects intracellular ROS level, was higher in wtHBx-transfected cells as compared with control cells. When wtHBx and HBxΔ127 were compared, the latter was found to induce more ROS in the transfected cells (Figure 4B). DHE stain was also used earlier (Hojo et al., 2017) to stain tumor cells. Therefore, the observation that HBxΔ127-transfected cells stain more intensely with DHE as compared with wtHBx is an important finding to implicate the truncation mutant in carcinogenesis.

### Tumor Initiation Clumps Observed in HBxΔ127-Transfected Cell Culture Plates

**FIGURE 5:**
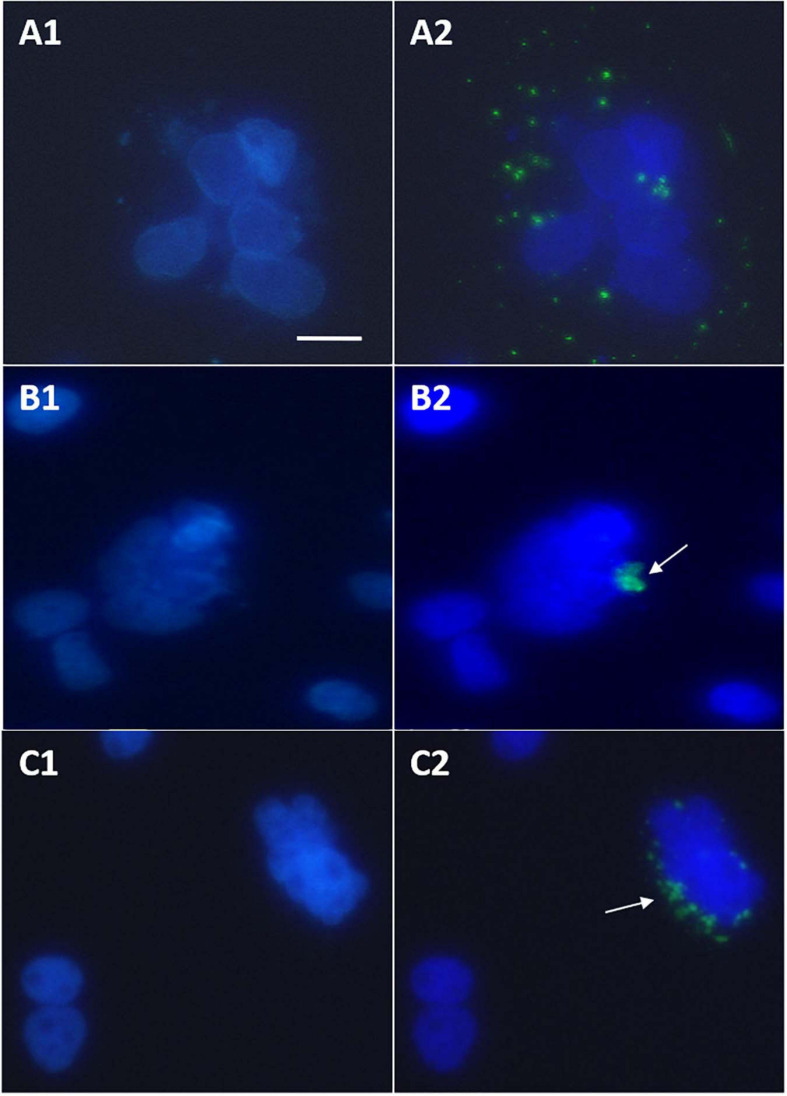
Huh7 cells cultured in 24-well plate and experiments performed at 70% confluency. Observations done after treating cells with DAPI antifade mountant 24 h post-transfection. **(A1,B1,C1)** Images showing DAPI-stained nuclei. **(A2,B2,C2)** Images showing overlap of **(A1,B1,C1**, respectively, with images of the same view field captured to observe GFP-HBx expression. **(A1,A2)** Loose aggregate of cells expressing GFP-HBx visible in cell cultures transfected with pEGFP-C3-HBx. **(B1,B2,C1,C2)** Tumor initiation clumps (TIC) visible in cell cultures transfected with pEGFP-C3-HBxΔ127. All observations done under inverted fluorescence microscope, 40× objective. Scale bar, 30 μm. Identical acquisition parameters were used while taking images. Refer to Supplementary Materials to see normal GFP expression and morphology of huh7 nuclei.

Microscopic observations of cell cultures transfected with GFP-wtHBx and GFP-HBxΔ127 revealed that some of the cells expressing the fusion proteins grow in clump or cluster form (Figure 5). The cells in the clumps formed in GFP-HBxΔ127 transfected cell cultures (Figures 5B,C) have properties of cancer cells (Hanahan and Weinberg, 2000) and named here as tumor initiation clumps (TICs). The cells in TIC have lost contact inhibition, they have a large nucleus-to-cytoplasm area ratio, and the cells are smaller as compared with neighboring cells not expressing GFP-HBxΔ127. Though some loose cell aggregates are also visible in GFP-wtHBx transfected cell cultures (Figure 5A), the cells in these aggregates look more like normal and not share the properties of cells present in TIC as mentioned previously. Further, the number of cell clumps in GFP-HBxΔ127-transfected culture wells was considerably higher than the number of loose cell aggregates found in GFP-wtHBx-transfected cultures.

### Wild-Type HBx Intracellular Expression Pattern

**FIGURE 6:**
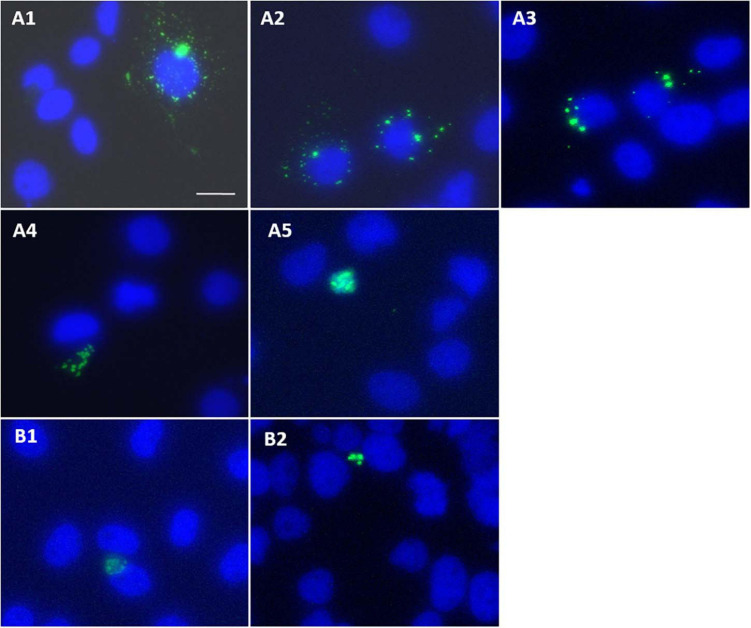
**(A1–A5)** Huh7 cells transfected with pEGFP-C3-HBx, and effect of post-transfection incubation time on expression pattern of GFP-HBx observed. Images captured 18–24 h **(A1,A2)**, 36–48 h **(A3)**, 48–72 h **(A4)**, 72–96 h, and onward **(A5)** post-transfection. **(B1,B2)** Expression of GFP-HBxΔ127 observed in cells transfected with pEGFP-C3-HBxΔ127, 18 h post-transfection. Expression of single granular body of GFP-HBxΔ127 at one pole of the nucleus observed as early as 18 h after cells were transfected with pEGFP-C3-HBxΔ127. Observations were done using an inverted fluorescence microscope, 40× objective. Scale bar, 30 μm. Identical acquisition parameters used while taking images.

Cells transfected with GFP-HBx showed perinuclear HBx expression as early as 18 h post-transfection (Figures 6A1, A2). Pattern of expression changed with course of time. At starting, GFP-HBx was expressed as fine perinuclear particles (Figure 6A1). Between 18 and 36 h, the particles increased in size and made an ordered ring around the nucleus (Figure 6A2). From 36 to 48 h, the particles coalesced, increasing in size, and decreasing in number, making the perinuclear ring more and more incomplete (Figure 6A3). As time elapsed, GFP-HBx particles started coalescing at one nuclear pole (Figures 6A3, A4), and finally all the transfected cells, at the end of around 96 h, were observed to have a single large granular body near the nucleus (Figure 6A5).

### HBxΔ127 Expression Pattern

It was observed that coalescing of expression granules into a single large granular body that was observed at around 96 h after transfection in case of GFP-wtHBx was achieved quite early in case of GFP-HBxΔ127. It seems that from the very beginning, that is around the time (18 h post-transfection) when GFP-wtHBx expression started showing up, GFP-HBxΔ127 expression was always observed in the form of a single large granular body near the nucleus (Figures 6B1, B2).

## Discussion

HBx is reported to be a crucial viral agent actively involved in liver cirrhosis and carcinogenesis in chronic hepatitis B patients (Feitelson et al., 2005). Because HBx is widely reported to contribute in carcinogenesis via promoting cell proliferation (Mukherji et al., 2007), the study started by simple MTT assay (Figure 2A) and flow cytometry analysis (Figure 2B) of wild-type (wt) and mutant HBx-transfected cells, to find out if the truncation mutant HBxΔ127 has an effect on the pro-growth/proliferation property of HBx (Mukherji et al., 2007). Increased distribution of cells to proliferating phases of cell cycle, observed in HBxΔ127-transfected cell population as compared with wtHBx, favors hepatocarcinogenesis as well as boosts HBV life cycle in the infected hepatocytes (Gearhart and Bouchard, 2010). Active viral turnover and increased multiplication rate of infected hepatocytes favor persistent HBV infection and chronicity. The results of cell growth assay (Figure 2C) further confirmed the promotion of cell growth/proliferation by HBxΔ127.

Transcription factor E2F regulates expression of genes that control cell cycle entry from G0/G1 into S phase. Examples include genes that code for DNA polymerase subunits, cyclin A and cyclin E (Sun et al., 2007). E2F1, a major subtype of E2F, is a positive regulator of cell proliferation. Overexpression of E2F1 is reported in HCC tumors as compared with non-tumor tissues (Nakajima et al., 2008). Higher expression of E2F1 in the presence of HBxΔ127 (as compared with wtHBx, Figure 3) indicates the role of this mutant in faster G1-S transition via upregulation of genes coding for DNA polymerase subunits, cyclin A and cyclin E. This underlines HBxΔ127 as an important contributor in proliferation and clonal selection of transformed cells, consistent with the results of flow cytometry (Figure 2B), cell growth assay (Figure 2C), and fluorescence microscopy (Figure 5).

B-Myb is an oncogenic transcription factor that upon CDK2-dependent phosphorylation stimulates transcription of late cell cycle genes (Werwein et al., 2019). B-Myb is upregulated in many cancers including HCC and is also a marker of poor prognosis in HCC patients. Overexpression of B-Myb is associated with cell proliferation, cell survival, differentiation, tumorigenesis, metastasis, and invasion (Musa et al., 2017). Therefore, upregulated B-Myb expression in the presence of HBxΔ127 (Figure 3) might be favoring host cell proliferation, as observed in the results of flow cytometry (Figure 2B), cell growth assay (Figure 2C), and fluorescence microscopy (Figure 5). Functionally activating B-Myb depends on its phosphorylation, which in turn depends on the expression level of CDK2. Higher expression of CDK2 (Figure 3) will increase the pool of activated B-Myb (Ziebold et al., 1997). Increased expression of B-Myb (Figure 3) may also be an effect of E2F1 overexpression (Figure 3), as B-Myb transcription is under control of E2F1 (Nakajima et al., 2008). Further, higher expression of E2F1, CDK2, and B-Myb (Figure 3) in HBxΔ127 harboring cells favor its proliferation (Figures 2B,C, and 5) and clonal selection, promoting HCC development. Moreover, it also highlights that even a slight upregulation in expression of genes involved in controlling cell growth/proliferation is worth noting, as these genes are interrelated and expression of one gene affects the expression of other genes.

C-Myb, another transcription factor, reported to be overexpressed in HCC, is involved in HBV-induced liver carcinogenesis, metastasis, and invasion (Chen et al., 2010). B-Myb and p27^Kip1^ are reported to be involved in HCC metastasis (Musa et al., 2017; Vinciguerra et al., 2019). Therefore, higher expression of B-Myb, C-Myb, and p27^Kip1^ observed in HBxΔ127 transfected cells (Figure 3) may contribute to the metastatic and aggressive attributes of HCC cells. Further, because C-Myb expression is controlled by E2F1 (Davis et al., 2005), the higher expression of C-Myb in the presence of HBxΔ127 might be a consequence of E2F1 overexpression (Figure 3).

Cell growth control by the tumor suppressor gene p27^Kip1^ is well reported (James et al., 2008). It is also known that p27^Kip1^ regulates actin cytoskeleton and cell migration (Vinciguerra et al., 2019). Increased expression of p27^Kip1^ contributes to cell cycle withdrawal and differentiation in advanced HCC stages (De Clercq and Inze, 2006). Expression of p27^Kip1^ was considerably higher in HBxΔ127 transfected cells as compared with wtHBx (Figure 3). This hints an important role of HBxΔ127 in HCC, via inducing expression of p27^Kip1^, in advanced stages of HCC, when the tumor cells acquire invasive property to kick-start the metastatic phase of cancer. This is in line with the fact that HBx truncations like HBxΔ127 are frequently present in chronic HBV patients suffering from HCC or its metastatic stages, and is not normally found in HBV patients not diagnosed with HCC (Liu et al., 2008; Ma et al., 2008; Zhang et al., 2008; Wang et al., 2010).

Upregulation of p21^Cip1^ has previously been related to cell proliferation in human liver diseases (Crury and Albrecht, 1998). In a study on chronically infected hepatitis C virus patients, p21^Cip1^ was found to be overexpressed in areas of liver damaged by oxidative stress and fibrosis (Wagayama et al., 2002). The authors predicted that upregulated p21^Cip1^ expression in liver cells contributes to HCC development via inducing oxidative stress. In addition, the role of p21^Cip1^ overexpression in carcinogenesis and poor prognosis of several cancers have been reported (Wang et al., 2019). Further, p21^Cip1^ as an oncogene had also been proposed in the context of HCC (Ohkoshi et al., 2015). Therefore, HBxΔ127 may contribute in HCC development and aggressiveness by inducing generation of intracellular ROS (Figure 4B) and p21^Cip1^ expression (Figure 3). Further, elevated p21^Cip1^ expression observed in the presence of HBxΔ127 (Figure 3) might be a direct consequence of higher ROS generation or vice versa (Figure 4B).

Healthy and actively growing cells have polarized mitochondria, whereas a depolarized mitochondrial population reflects damaged/dysfunctional mitochondria and poor cell health (Sivandzade et al., 2019). HBxΔ127 may contribute in initial stages of HCC development via maintaining a more polarized, that is, more functional/healthy, mitochondrial population (Figure 4A) required for actively proliferating tumor cells.

Elevated ROS level maintained in liver cells of chronically infected HBV patients is a key player for HBV-related disease development (Guicciardi and Gores, 2005; Hu et al., 2011). Expression of HBx in HBV-infected hepatocytes is known to cause ROS production (Ren et al., 2016). High level of ROS causes mitochondrial damage and cell necrosis, directly contributing to hepatic inflammation and cirrhosis. High level of ROS is also reported to cause fibrosis in hepatic tissue (Cichoż-Lach and Michalak, 2014). Further, DHE stain that is used for detecting intracellular ROS in this study has also been used to stain tumor cells (Hojo et al., 2017). Therefore, HBxΔ127 might be favoring hepatic fibrosis, cirrhosis, and HCC development by inducing ROS production (Figure 4B) in the infected host hepatocytes.

The TIC formation observed in GFP-HBxΔ127-transfected cell cultures (Figure 5) might be a direct consequence of high cell growth/proliferation (Figures 2B,C), and upregulated expression of CDK2, B-Myb, C-Myb, and E2F (Figure 3) in presence of HBxΔ127. Considering the fact that HBx C-terminal truncations, a representative example of which is HBxΔ127, are frequently expressed in chronic HBV patients suffering from HCC and not common in chronic HBV patients not suffering from cirrhosis and HCC (Liu et al., 2008; Ma et al., 2008; Zhang et al., 2008; Wang et al., 2010; Ali et al., 2014; An et al., 2018), the truncated version of HBx seems to be directly involved in tumorigenesis of the infected hepatocytes (Figure 5).

A proportion of intracellular expressed HBx is localized on mitochondria, primarily because of HBx direct interaction with voltage-dependent anion channels on mitochondrial membrane. This leads to change in transmembrane mitochondrial potential (Rahmani et al., 2000) and perinuclear mitochondrial clustering (Kim et al., 2007). In addition, cells producing high level of ROS show reorganization and clustering of mitochondria around nuclear periphery, and perinuclear localization is directly related to ROS level (Al-Mehdi et al., 2012). Large intracellular aggregates of HBx are often found in cells overexpressing HBx (Song et al., 2003). The ring like perinuclear localization of GFP-HBx (Figure 6A1) seen as early as 18 h post-transfection is mainly because of HBx mitochondrial localization and resulting mitochondrial aggregation (Rahmani et al., 2000; Kim et al., 2007). As time passes after transfection, HBx-harboring bodies become less numerous and larger in size, and the ring becomes more and more incomplete because of gradual mitochondrial aggregation, finally to make a single GFP-HBx harboring mitochondrial aggregate body (Figures 6A1–A5). The early formation of single aggregated body (Figures 6B1,B2) might be a consequence of higher polarized mitochondria (Figure 4A) and higher level of intracellular ROS (Figure 4B) in HBxΔ127 expressing cells. Such redistribution and clustering of mitochondria are predicted to be important for transcriptional activation of genes involved in pathological conditions (Al-Mehdi et al., 2012).

Though a few reports are available on the truncation mutant HBxΔ127 (Zhang et al., 2008; Wang et al., 2010; Ali et al., 2014), our findings are novel in many ways. The effect of the truncation mutant on expression of E2F1, B-Myb, C-Myb, CDK2, p21^Cip1^, and p27^Kip1^, and its possible involvement in HCC development and metastasis would be a significant addition to the existing knowledge. The impacts of the truncation mutant on mitochondrial potential and intracellular ROS, and possible consequences on liver fibrosis, cirrhosis, and carcinogenesis have never been reported. Direct observation of the formation of tumor-like clumps (TIC) with the help of GFP fusion protein in transiently transfected cell cultures is new and valuable information. Although the perinuclear clustering of mitochondria in cells expressing HBx is well known (Takada et al., 1999; Kim et al., 2007), change of expression pattern in a time-dependent manner, up to the formation of single aggregate body of HBx–mitochondria, is not reported earlier. Hastened formation of HBx–mitochondria single aggregate body at nuclear periphery in cells expressing GFP-HBxΔ127 is a new phenomenon. Considering the fact that there are several C-terminal truncations of HBx frequently expressed in HBV-related cirrhosis and HCC patients (Ma et al., 2008; An et al., 2018), and out of these, HBxΔ127 is a common example (Liu et al., 2008; Wang et al., 2010; Ali et al., 2014), our findings are important in understanding how the expression of a truncated version of HBx can affect molecular and cellular processes, and contribute toward the development of HBV-related liver diseases.

### Limitations

This study used only huh7 cell line, so further studies using other cell lines should be performed to consolidate the novel findings of this study. Further, in depth studies on gene expression should be undertaken to further explore the findings of real-time RT-PCR using Western blot or flow cytometry.

## Data Availability Statement

The original contributions presented in the study are included in the article/Supplementary Material, further inquiries can be directed to the corresponding author.

## Author Contributions

ZS conceived, planned, and executed the experiments. SA, MA, and AA supported and assisted in experiments. WK helped in cloning experiments and cell culture. SF guided in experiments and literature review. SP supported in PCR facilities. FA, MA, SK, and MM assisted in analyzing the results. SNK supervised all the experiments. All authors discussed the results and contributed to the final manuscript.

## Conflict of Interest

The authors declare that the research was conducted in the absence of any commercial or financial relationships that could be construed as a potential conflict of interest.
