# Supplementary material for: An in vitro Study on the Role of Hepatitis B Virus X Protein C-Terminal Truncation in Liver Disease Development
Source: Front Genet. 2021 Mar 12;12:633341. doi: 10.3389/fgene.2021.633341 (PMC7994528; doi:10.3389/fgene.2021.633341)
Supplement: Supplementary file 1 [file Data_Sheet_1.pdf]

Supplementary materials

Name of primer	Sequence
**Cloning primers**	
HBx-KpnI-F	5´CAGGTACCATGGCTGCTAGGCTGT3´
HBx-XhoI-R	5´GCCTCGAGTTAGGCAGAGGTGAAAAAGTT3´
**Sequencing primers**	
T7 promoter forward	5’TAATACGACTCACTATAGGG3’
BGH reverse	5’TAGAAGGCACAGTCGAGG3’
EGFP C	5’CATGGTCCTGCTGGAGTTCGTG3’
**SDM primers**	Substituted nucleotides at the mutation site highlighted bold
HBxΔ127-F	5´GGAGTTGGGGGAGGAGATT**TA**GTTAAAGGTCTTTGTACTAG3´
HBxΔ127-R	5´CTAGTACAAAGACCTTTAAC**TA**AATCTCCTCCCCCAACTCC 3´
**RT-PCR primers**	
HBx-F	5´GCTGCTAGGCTGTACTGC3´
HBx-R	5´TTAGGCAGAGGTGAAAAAG3´
**Real time qRT-PCR primers**	Annealing temperature for the primer set in bracket
cdk2-F	5´GCTAGCAGACTTTGGACTAGCCAG3´ (59ºC)
cdk2-R	5´AGCTCGGTACCACAGGGTCA3´
b-myb-F	5´AGAGGGATAGCAAGTGCAAGGT3´ (58ºC)
b-myb-R	5´TGTACTGGCATTGCTGGTCAGT3´
c-myb-F	5´TACAATGCGTCGGAAGGTCG3´(55ºC)
c-myb-R	5´GCGGAGCCTGAGCAAAACC3´
E2F1-F	5´AGATGGTTATGGTGATCAAAGCC3´(52ºC)
E2F1-R	5´ATCTGAAAGTTCTCCGAA GAGTCC3´
p21-F	5´TGAGCCGCGACTGTGATG3´(65.5ºC)
p21-R	5´GTCTCGGTGACAAAGTC GAAGTT3´
p27-F	5´CTGCAACCGACGATTCTTCTACT3´(64ºC)
p27-R	5´GGGCGTCTGCTC CACAGA3´
GAPDH-F	5´ATGACCCCTTCATTGACC3´
GAPDH-R	5´GAAGATGGTGATGG GATTTC3´(58ºC)

1. Some peer reviewed research articles are presented below as examples to show that pcDNA3 and pEGFP-C3 are commonly used expression vectors for *in vitro* study of HBV infection and liver disease processes.

Tian Y, Xiao X, Gong X, Peng F, Xu Y, Jiang Y, Gong G. HBx promotes cell proliferation by disturbing the cross-talk between miR-181a and PTEN. Sci Rep. 2017; 7: 40089.

Fu X, Song X, Li Y, Tan D & Liu G. (2016). Hepatitis B virus X protein upregulates DNA methyltransferase 3A/3B and enhances SOCS-1CpG island methylation. Molecular Medicine Reports, 13(1), 301-8.

Kong, F., Hu, W., Zhou, K. et al. Hepatitis B virus X protein promotes interleukin-7 receptor expression via NF-κB and Notch1 pathway to facilitate proliferation and migration of hepatitis B virus-related hepatoma cells. J Exp Clin Cancer Res 35, 172 (2016).

Chen, S.L., Liu, L.L., Lu, S.X., Luo, R.Z., Wang, C.H., Wang, H., Cai, S.H., Yang, X., Xie, D., Zhang, C.Z. & Yun, J.P. (2017). HBx-mediated decrease of AIM 2 contributes to hepatocellular carcinoma metastasis. Molecular Oncology, 11(9), 1225-40.

Kongkavitoon P, Tangkijvanich P, Hirankarn N, Palaga T (2016) Hepatitis B Virus HBx Activates Notch Signaling via Delta-Like 4/Notch1 in Hepatocellular Carcinoma. PLoS ONE 11(1): e0146696.

Han, J., Ding, L., Yuan, B., Yang, X., Wang, X., Li, J., Lu, Q., Huang, C. & Ye, Q. (2006). Hepatitis B virus X protein and the estrogen receptor variant lacking exon 5 inhibit estrogen receptor signaling in hepatoma cells. Nucleic Acids Research, 34(10), 3095-106.

2. Huh7 cell line has been used extensively by the scientific community for the *in vitro* study of liver diseases, specifically HCC. We have presented here the following research/review articles to show that huh7 cell line is a suitable cell line for this study.

Xu J, Ji B, Wen G, Yang Y, Jin H, Liu X, Xie R, Song W, Song P, Dong H and Tuo B. Na+/H+ exchanger 1, Na+/Ca2+ exchanger 1 and calmodulin complex regulates interleukin 6-mediated cellular behavior of human hepatocellular carcinoma. Carcinogenesis 2016; 37(3):290-300.
Cell line used: Huh7

Rajput P, Shukla SK and Kumar V. The HBx oncoprotein of hepatitis B virus potentiates cell transformation by inducing c-Myc-dependent expression of the RNA polymerase I transcription factor UBF. Virology Journal 2015; 12:62.
Cell line used: Huh 7 (hepatoma derived) and IHH (hepatocyte derived) compared for each assay and comparable results found.

Damania P, Sen B, Dar SB, Kumar S, Kumari A, et al. Hepatitis B Virus Induces Cell Proliferation via HBx-Induced microRNA-21 in Hepatocellular Carcinoma by Targeting Programmed Cell Death Protein4 (PDCD4) and Phosphatase and Tensin Homologue (PTEN). Plos ONE 2014; 9(3):e91745.
Cell line used: HepG2, Huh7 and HepG2.2.15

Na TY, Ka NL, Rhee H, Kyeong D, Kim MH, Seong JK, Park YN, Lee MO. Interaction of hepatitis B virus X protein with PARP1 results in inhibition of DNA repair in hepatocellular carcinoma. Oncogene 2016; 35(41):5435-5445.
Cell line used: Chang cells, HepG2, Huh7 and Chang X-34 cells.

Zheng BY, Fang XF, Zou LY, Huang YH, Chen ZX, Li D, Zhou LY, Chen H and Wang XZ. The co-localization of HBx and COXIII upregulates COX-2 promoting HepG2 cell growth. International Journal of Oncology 2014; 45:1143-1150.
Cell line used: HepG2 (hepatoma derived similar as Huh7 cell line) used for cell growth study

Vijaya Pandey & Vijay Kumar. Stabilization of SIRT7 deacetylase by viral oncoprotein HBx leads to inhibition of growth restrictive *RPS7* gene and facilitates cellular transformation. Scientific Reports 2015; 5:14806.

Cell line used: Huh7 used for cell invasion and migration assay

Yang, Haung YS, Chao TW, Tsai TF and Su IJ, Hsieh WC, Ching-Wen. Chemoprevention of HBV-related hepatocellular carcinoma by the combined product of resveratrol and silymarin in transgenic mice. Functional Foods in Health and Disease 2013; 3(9):341-352.
Cell line used: Huh7 used for cell proliferation study

Liu J, Ding X, Tang J, Cao Y, Hu P, et al. Enhancement of Canonical Wnt/b-Catenin Signaling Activity by HCV Core Protein Promotes Cell Growth of Hepatocellular Carcinoma Cells. PLoS ONE 2011; 6(11):e27496.
Cell line used: Huh7 used for cell proliferation and cell cycle progression studies.

Slagle BL, Andrisani OM, Bouchard MJ, Lee CGL, Ou JHJ, and Siddiqui A. Technical Standards for Hepatitis B Virus X protein (HBx) Research. Hepatology 2015; 61(4): 1416-1424.
Huh7 and HepG2 are most commonly used for HBx oncogenic studies

Krelle AC, Okoli AS, Mendz GL. Huh-7 Human Liver Cancer Cells: A Model System to Understand Hepatocellular Carcinoma and Therapy. Journal of Cancer Therapy 2013; 4:606-631.

3. Control experiments for DAPI staining and GFP expression.

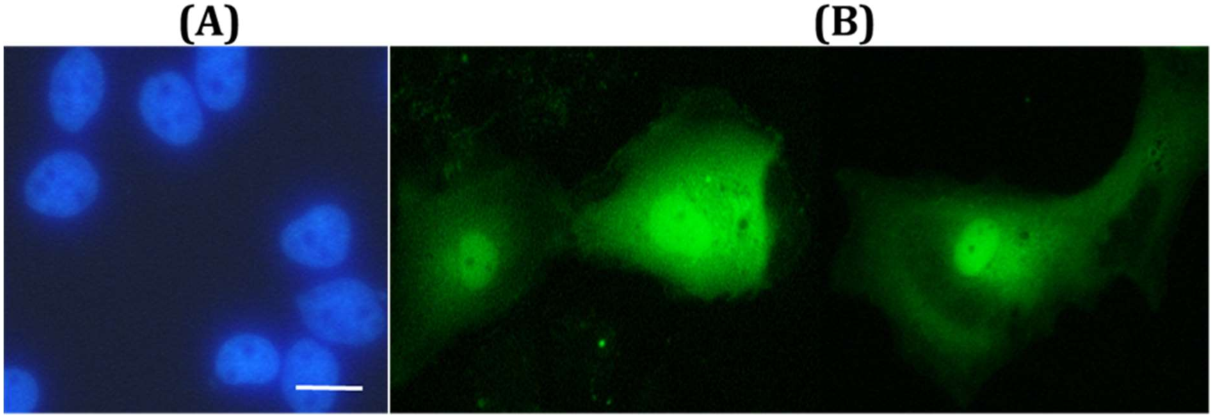

Figure. Huh7 cells cultured in 24-well plate and transfection performed at 70% confluency. **A,** Healthy cells treated with DAPI antifade mountant to show morphology of nuclei under normal cell growth conditions. **B,** Cells transfected with empty pEGFP-C3 and observed 24 hours post-transfection to observe normal GFP expression. Scale bar, 30μm.

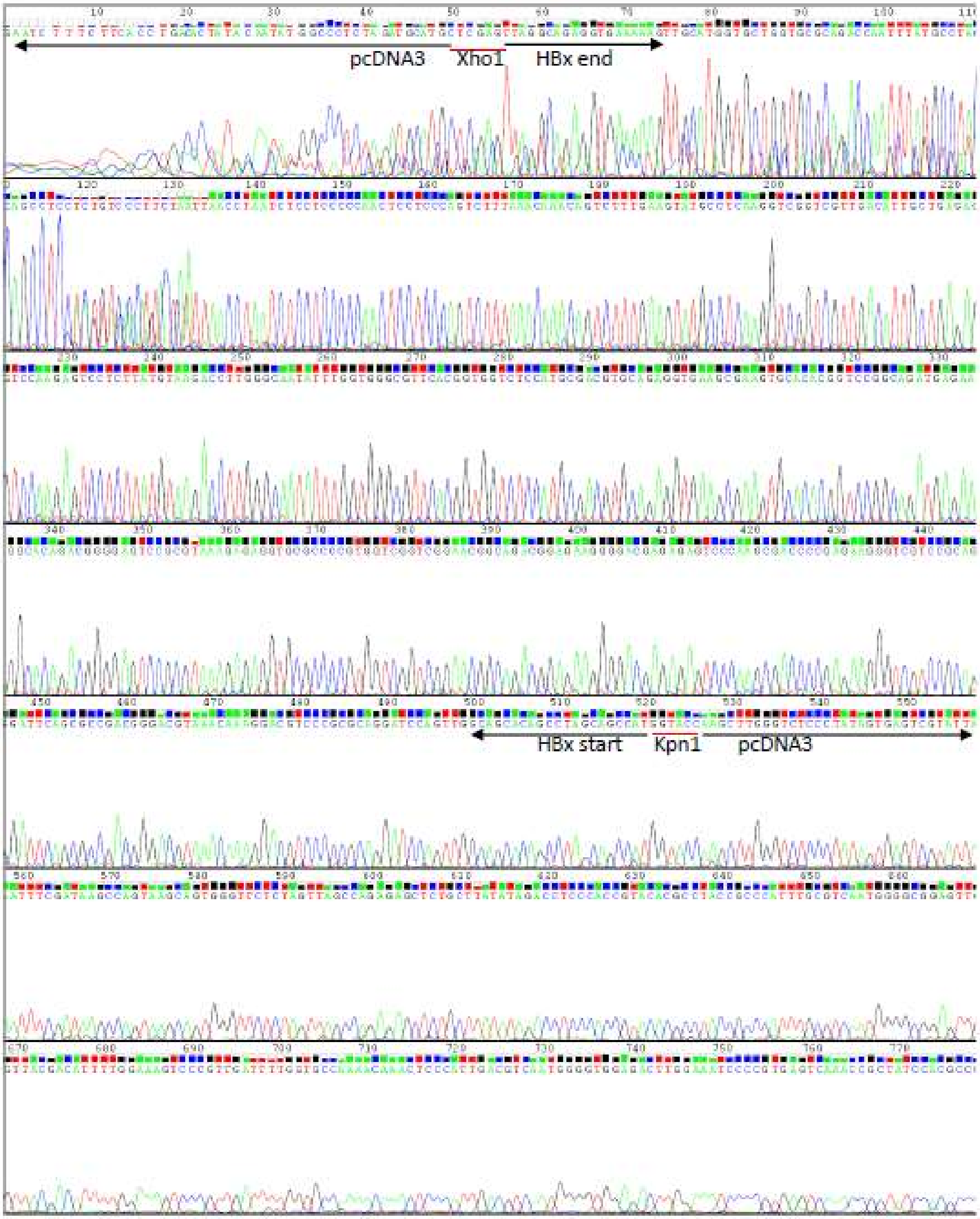

Electrophorogram 1, obtained after sequencing pcDNA3-HBx with BGH reverse primer. Arrows indicate the integration sites of HBx insert and the vector. Restriction sites used for cloning are underlined red.

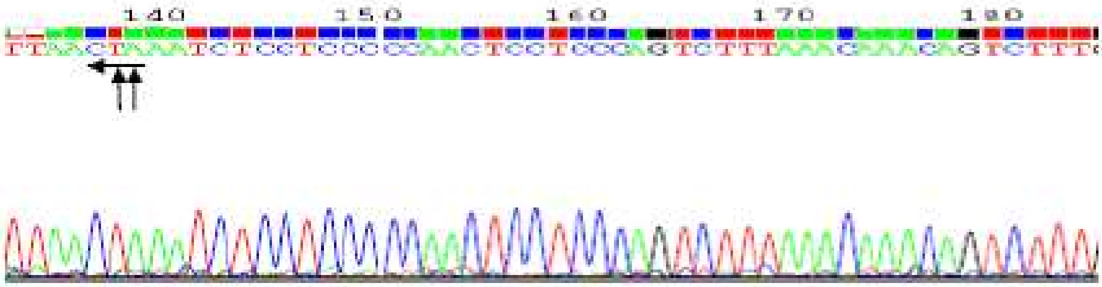

Electrophorogram 2, showing the mutation site for HBxΔ127 in the construct pcDNA3-HBxΔ127. The codon **AG**G for arginine at amino acid 128th of HBx is converted to stop codon **UA**G, resulting in truncation of last 27 amino acids of HBx. Sequencing done by BGH reverse primer.

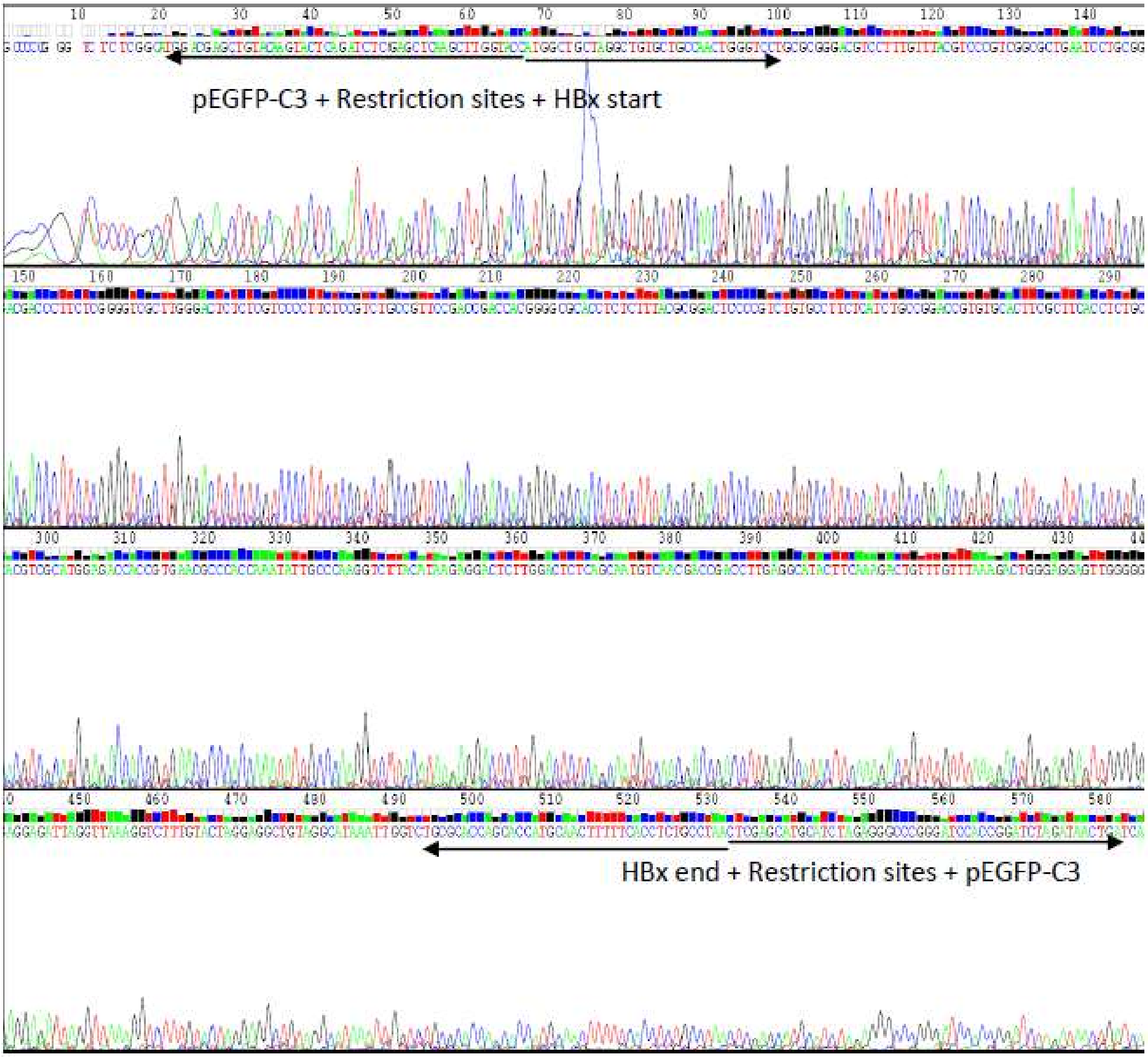

Electrophorogram 3, for the construct pEGFP-C3-HBx. The start and end of HBx gene, the restriction sites involved, and the flanking regions of the vector are marked by arrows. Sequencing done by EGFP C primer.

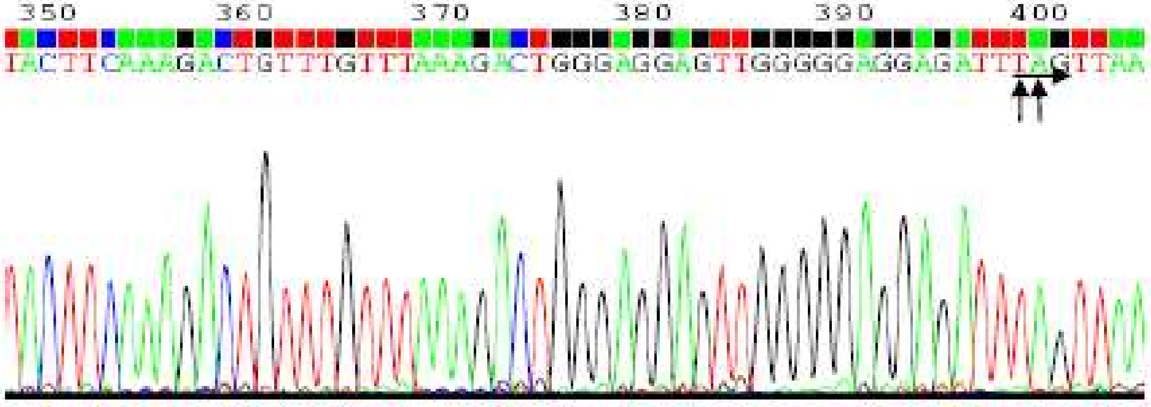

Electrophorogram 4, showing the mutation site for HBxΔ127 in the construct pEGFP-C3-HBxΔ127. The codon **AG**G for arginine at amino acid 128th of HBx was successfully converted to stop codon **UA**G, resulting in truncation of last 27 amino acids of HBx. Sequencing done by EGFP C primer.

Reagent/Chemical/Machine/instrument	Company/Brand	Catalogue No/Machine Serial/Product No.
Acrylamide	Sigma-Aldrich	A9099
Anti-HBx antibody	Santa Cruz Biotechnology	sc-78239
Anti-GAPDH antibody	Thermo Scientific	14-9523-95
ApaI	Thermo Scientific	ER-1411
Aprotinin	Sigma-Aldrich	A6106
Bisacrylamide	Sigma-Aldrich	294381-25G
Bovine serum albumin	G-Biosciences	A3311-100G
Crystal violet	Sigma-Aldrich	1159400025
DAPI antifade mountant	Santa Cruz biotechnology	sc-3598
Dihydroethidium	Sigma-Aldrich	37291
Dimethyl sulfoxide	Sigma-Alidrich	D8418-100 ml/ D2650
DpnI	Thermo Scientific	ER-1701
ELISA microplate reader	Bio-Rad	S/N: 12885
Enhanced chemiluminescence reagents	Thermo Scientific	34579
Fetal bovine serum	Genetix Biotech	CCS-500-SA-U
Goat anti-mouse HRP conjugated IgG antibody	Santa Cruz Biotechnology	sc-2060
High fidelity Taq DNA polymerase	Thermo Scientific	K0192
HindIII	Thermo Scientific	ER-0501
InsTAclone PCR Cloning Kit	Thermo Scientific	K1214
Inverted fluorescent microscope	Nikon	4N75 Model No. Ti-S
KpnI	Thermo Scientific	ER0521
Laemmli buffer	Amresco	M337
Lipofectamine 2000	Invitrogen	11668030
MTT	Sigma-Alidrich	M2128
Nitrocellulose membrane, BioTrace NT	Pall Corporation	66485
PCR machine	Applied Biosystems	Model No. 9902
Phenylmethylsulfonyl fluoride	Sigma-Alidrich	P7626
Propidium Iodide	Merck	537059
Penicillin-Streptomycin	Genetix Biotech	SV30010
Protein Estimation Kit by BCA Method	GeNei™	SKU: *KT31*
RIPA buffer	Thermo Scientific	89900
Reverse transcriptase (SuperScript™ IV)	Thermo Scientific	18090010
RNaseA	Merck	10109142001
RNase inhibitor	Thermo Scientific	N8080119
Sodium dodecyl sulfate	Sigma-Alidrich	L3771
Sodium fluoride	Sigma-Aldrich	S7920
Sodium orthovanadate	Sigma-Aldrich	13721-39-6
StepOnePlus real-time PCR machine	Applied Biosystems	S/N: 2720031917

Sure Extract PCR Clean-up/Gel Extraction Kit	Genetix Biotech	NP-36105
SYBR Green I	Applied Biosystems	A25742
T4 DNA ligase	Thermo Scientific	EL0011
Tetramethylrhodamine ethyl ester	Sigma-Aldrich	87917
TRIzol® Reagent	Invitrogen	15596-018
Tween-20	Merck	SK0S600743
XhoI	Thermo Scientific	ER-0692
